# Specific IgEs passively transferred through a platelet transfusion caused two discrete allergic reactions to food

**DOI:** 10.1186/1710-1492-10-S1-A18

**Published:** 2014-03-03

**Authors:** Joyce CY Ching, Wendy Lau, Barbara Hannach, Julia EM Upton

**Affiliations:** 1 Department of Pediatrics, Rouge Valley Health System, Toronto, Ontario, Canada, M1E 4B9; 2 Transfusion Medicine, Pediatric Laboratory Medicine, University of Toronto, Hospital for Sick Children, Toronto, Ontario, Canada, M5G 1X8; 3 Canadian Blood Services, Toronto, Ontario, Canada, M5G 2M1; 4 Department of Pediatrics, Division of Clinical Immunology and Allergy, University of Toronto, Hospital for Sick Children, Toronto, Ontario, Canada, M5G 1X8

## Background

An unusual cause of an allergic reaction to food is the passive transfer of specific IgE through blood product transfusions. We report two separate allergic reactions due to passive transfer of food specific IgE from a pooled platelet transfusion.

## Case report

A non-atopic 8 year old boy received multiple blood product transfusions as part of his treatment for meduloblastoma. He subsequently experienced anaphylaxis to salmon. Within minutes of eating salmon, he developed angioedema of the lip, facial erythema, throat discomfort and low blood pressure. Before this episode, he regularly ate fish with no reaction. The passive transfer of food specific IgE was suspected and he was advised to carry an epinephrine auto-injector and avoid all vertebrate fish. Specific IgE to salmon by ImmunoCAP was positive. Follow-up was arranged to follow his specific IgE to salmon with the expectation that his allergy would resolve. One week after his anaphylactic episode to fish, he developed an allergic reaction to peanuts. He ate a chocolate peanut butter cup and within 10 minutes he vomited, developed angioedema of the lip and experienced lethargy. Previously, he routinely ate peanuts without any symptoms. Skin prick testing showed positive results to peanut, salmon, mixed fish, and tree nut mix. He had a positive ImmunoCAP to peanut. Approximately 6 months later, he had undetectable ImmunoCAP results to both salmon and peanut. He resumed consumption of salmon and peanuts with no reaction. As part of the adverse event investigation by Canadian Blood Services all donors associated with the reaction were contacted and one donor stated that they have a severe allergy to peanuts, tree nuts, shellfish, and all fish including salmon. This information implicated one specific pooled platelet transfusion in which the platelets were suspended in the plasma of the atopic donor. The donor has been excluded from future donations.

## Conclusions

To our knowledge, this is the first reported case of two allergic reactions to food documented to be caused by passive transfer of food-specific IgE from pooled platelets. This case shows that if a passive allergy from a transfusion occurs, consideration should be given to look for additional passively transferred specific IgE. Knowledge of the allergies in the blood donors can assist in the investigations and avoidance instructions.

